# Skeletal fluorosis in relation to drinking water in rural areas of West Azerbaijan, Iran

**DOI:** 10.1038/s41598-017-17328-8

**Published:** 2017-12-11

**Authors:** Ali Akbar Mohammadi, Mahmood Yousefi, Mehdi Yaseri, Mohsen Jalilzadeh, Amir Hossein Mahvi

**Affiliations:** 1 Department of Environmental Health Engineering, Neyshabur University of Medical Sciences, Neyshabur, Iran; 2 Department of Environmental Health Engineering, School of Public Health, Tehran University of Medical Sciences, Tehran, Iran; 3 Department of Epidemiology and Biostatistics, School of Public Health, Tehran University of Medical Sciences, Tehran, Iran; 4 Department of Epidemiology, Urmia University of Medical Sciences, Urmia, Iran; 5 Center for Solid Waste Research, Institute for Environmental Research, Tehran University of Medical Sciences, Tehran, Iran

## Abstract

Skeletal fluorosis resulting from high fluoride level in drinking water is a major public health problem. The present study evaluated the association between exposures to drinking water fluoride and skeletal fluorosis in 5 villages of Poldasht County, Iran. All the data and information on the prevalence of bone diseases were obtained from the Health Record Department, Poldasht Health Centre. To obtain the odds ratio of bone disease problem in different risk factors, when considering the cluster effect of rural area, logistic regression in a multilevel model was used. Results showed that skeletal fluorosis of people who live in areas with high fluoride concentration is 18.1% higher than that of individuals who live in areas with low fluoride concentration. Skeletal fluorosis (54.5%) was observed in the age group of 71 years and above, and was more commonly found in females than males. According to Unadjusted, individuals who consume ≤3 unit milk and dairy products per week have almost the same level of bone diseases as compared to those that consume more than 3 units. This study indicated that, skeletal fluorosis is a general health problem in these rural areas because the results revealed that high percentage of the studied population had symptoms of skeletal fluorosis.

## Introduction

Fluoride is one of the anions that endanger human health at concentrations lower and higher than the standard, and this is one of the main problems in most parts of the world. About 200 million people from 25 countries are exposed to high concentrations of fluoride from groundwater sources^1,2^. Fluoride is an element from the halogen group and its average concentration in the Earth’s crust is 0.3 kg, while its background concentration in the atmosphere is 3 ng per m^2^. Fluoride enters into water sources from natural resources, as well as industries where the mineral fluoride is used as raw material for extraction of aluminum, mining, pottery, making of ceramics, bricks, and manure which are the artificial and important contamination sources of fluoride^3–6^. The relationship between fluoride and human health was first discussed in the late nineteenth century when chemists observed different concentrations of fluorine in the tissues, bones and teeth of human^7,8^. Fluoride is one of the essential micro-elements for animals and humans and its consumption at a reasonable range protects teeth against microbial attack, especially in childhood; however, exposure to excessive concentration of fluoride can damage skeletal tissue (bones and teeth). However, fluoride minimum nutritional requirement cannot be exactly determined but the risk of adverse effects on the skeleton can be observed at concentrations higher than 6 mg/l per day^9–16^. Fluoride is absorbed by the human body through food, drinking water, toothpaste, mouthwash products and air, but air cannot be a major source for absorption of this element; however, the gastrointestinal tract is one of the main routes through which fluoride is absorbed and food is the most important source. Fluoride can be found in every type of food, but some foods like curly kale, endive and fish have higher fluoride content than others. Among the listed sources for fluoride absorption, drinking water is the main one in most societies^2,3^. Fluoride is variable in water sources and mainly depends on the type of stone and soil which water flows from. Many epidemiological studies have shown that absorption of fluoride in drinking water in the long term leads to adverse effects on the human skeletal tissue. Therefore, the World Health Organization (WHO) has considered the minimal concentration of 1.5 mg/L^2^ ^17,18^. Worldwide, the concentration of fluoride in drinking water and its relation to bone and skeletal diseases have been investigated in some restricted areas^19–22^. Thus, the aim of the present study was to investigate the prevalence of skeletal diseases among people in two municipalities that had different standards with regards to control of the optimal fluoride concentration in water consumed by the population.

## Materials and Methods

### Study areas

Poldasht county is located in North West of Azerbaijan province, Iran with coordinates (UTM) X = 446625 to 513055 to the east and Y = 4344280 to 4402863 to the north. Poldasht meteorological station showed that in a long-term, the average rainfall is equal to 131.5 mm. The city also has borderline in the West and North with Turkey. Two study areas (five villages) were selected in Poldasht County with almost the same socioeconomic status and dietary habits but different natural concentrations of F in drinking water. Two villages had a high level of F (Sari su, Konikor, Ag otlogh) and two had low level of F (Hasan kandi and Shiblu).

### Determination of the water fluoride concentration

Four drinking water wells in the area were selected. A total of 60 samples were obtained over three consecutive years, 2013 to 2015. The water samples were collected from ground water wells in sterile plastic 2-L container, then transported to the Laboratory for Water and Sewage, Poldasht. Fluoride concentration of the samples was determined using SPADNS method according to the Standard. The samples of the ground water were analyzed for physico-chemical parameters: total hardness (TH), chloride, nitrate, electrical conductivity, total alkalinity (TA), total dissolved solids (TDS), bicarbonate (HCO^3−^) and fluoride, using standard analytical methods^23^.

### Data collection

All data and information on prevalence of bone diseases were obtained from the Health Record Department, Poldasht Health Centre on the areas investigated. Based on this information given as health cases or questionnaire sheets, 445 persons in the high F area and 470 in the low F area, were identified.

Skeletal fluorosis was assessed using three simple diagnostic tests:

- 1- Touching the toes without bending the knees
- 2- Touching the chest with the chin; and stretching
- 3- The arms placed sideways and folding them to touch the back of the head^24^.

If there is pain or stiffness in the backbone, hip and joints; neck; shoulder joint and backbone, respectively, these three exercises cannot be performed.

### Statistical method

To present the data, mean, standard deviation, median and range were used. To compare the case and control groups, Chi-square and Mann-Whitney tests were used. To obtain the odds ratio of bone disease problem in different risk factors, when considering the cluster effect of rural area, logistic regression in a multilevel model was used. In the last step, the simultaneous effect of all risk factors on bone disease was evaluated by multiple multilevel logistic regression. All statistical analyses were performed by STATA (version 14). P-value less than 0.05 was considered to be statistically significant.

## Results

### Physico-chemical characteristics

**Figure 1:**
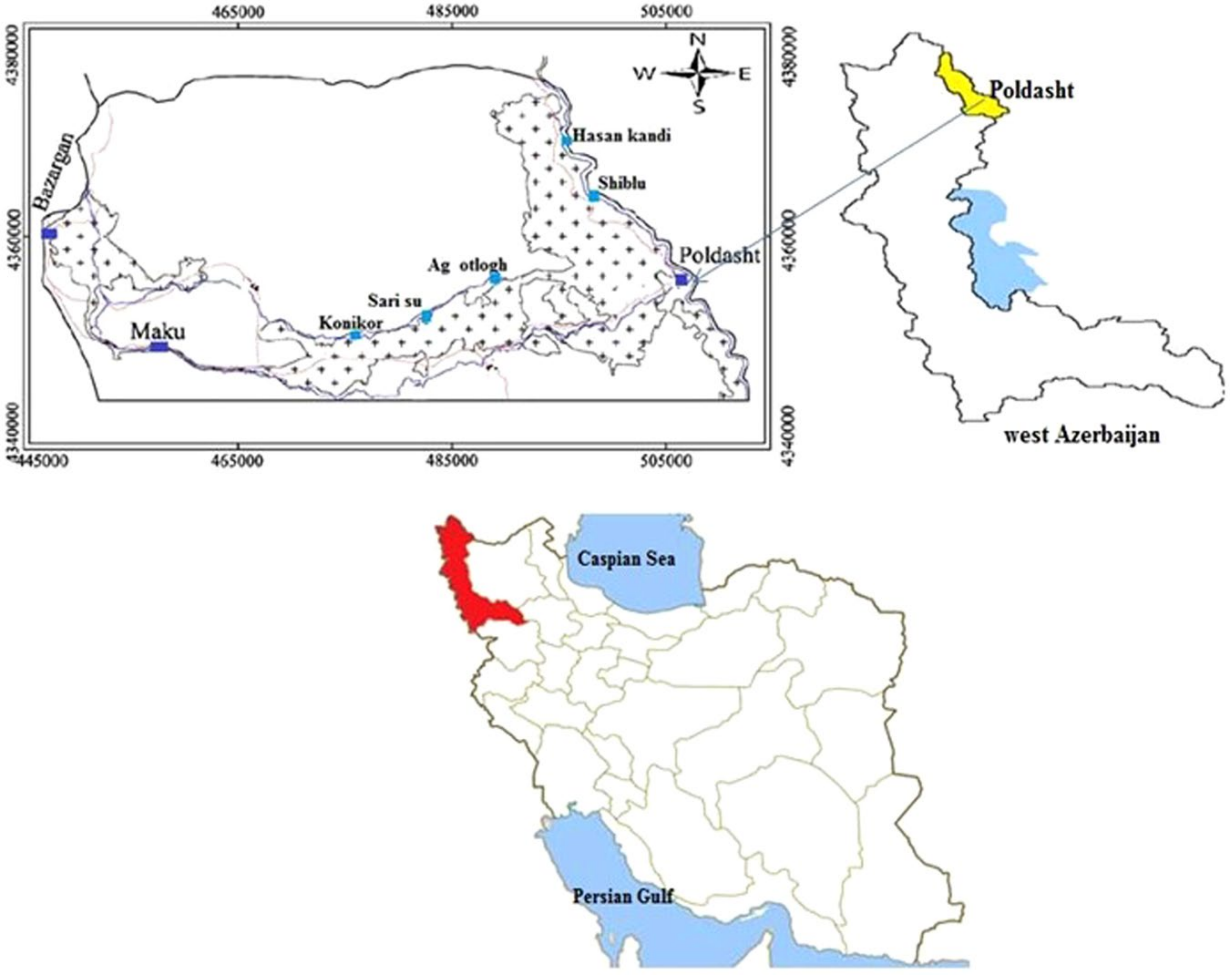
Location of the study areas in Poldasht City, West Azerbaijan, Iran^36^.

Data presented here shows the monitoring of physical and chemical characteristics including pH, EC, TDS, HCO_3_, CO_3_,SO_4_, Cl, NO_3_ and F in Poldasht County, West Azerbaijan, Iran. Figure 1 shows the study area and the sampling points. A summary of water quality characteristics and correlation of the parameters with fluoride are presented in Tables 1 and 2, respectively. Fluoride ion varied from 0.68 to 10.30 mg/L. This is given in Tables 1 and 2. Minimum (0.68 mg/L) and maximum (10.3 mg/L) concentrations of F were observed in Hasan kandi and Agh otlogh villages, respectively. Also, according to the data, 45% of villagers are in the desirable range and in 55 of them, the amount of fluorine was more than the standard level.

**Table 1:** Average of physico-chemical parameters of drinking water samples in the study areas.

Chemical parameters	n	Village					W.H.O. Guide line
		Sarisu	Hasan kandi	Shiblou	Agh otlogh	Konikor	
pH	60	7.48	7.76	7.32	7.68	7.76	6.5–8.5
EC (µS/cm)	60	1523.6	1484.4	1960	1059.2	1561.8	
TDS (mg/L)	60	591	724.2	948	740.2	1012.8	500
Nitrate (mg/L)	60	7.63	5.62	3.9	2.1	20.48	10
So4 (mg/L)	60	70.6	240	202	101	77.2	200
Total hardness (mg/L)	60	173.8	449.6	417	169	243	200
chloride (mg/L)	60	48	125	235.4	47	44.1	200
HCO_3_− (mg/L)	60	773.6	379.2	384	761.4	770.4	−
F (mg/L)		7.63	0.68	0.79	10.15	4.02	1.5–2

**Table 2:** Fluoride concentration in drinking water from 2013–2015 in the study areas.

	Mean	Normal	High
	Median	0.90	7.85
	Standard Deviation	0.91	7.60
Fluoride	Range	0.22	1.50
	Minimum	0.68	4.30
	Maximum	0.49	6.00
	Range	1.17	10.30

^*^P-values < 0.05, **P-value < 0.01.

### Correlation of fluoride concentration with other physicochemical parameters

In order to study the possible relationship between fluoride concentration and other physicochemical parameters studied, correlation studies were performed. There was a positive correlation between fluoride level and HCO_3_ (r = 0.646, P = 0.001) as compared to other physicochemical parameters studied. Other parameters such as PH, EC, TDS, ALK, chloride, nitrate and sulfate had a weak correlation with fluoride (Table 3).

**Table 3:** Spearman correlation of the parameters with fluoride.

	r	R-Square	P-value
HCO3	0.646**	0.417	0.001
SO4	−0.543**	0.295	0.005
CL	−0.692**	0.479	<0.001
NO3	−0.498*	0.248	0.013
ALK	0.546**	0.298	0.005
TH	−0.866**	0.750	<0.001
EC	−0.133	0.018	0.527
TDS	−0.015	0.000	0.942
pH	0.035	0.001	0.867

### Comparison of various epidemiological factors influencing the skeletal fluorosis

**Figure 2:**
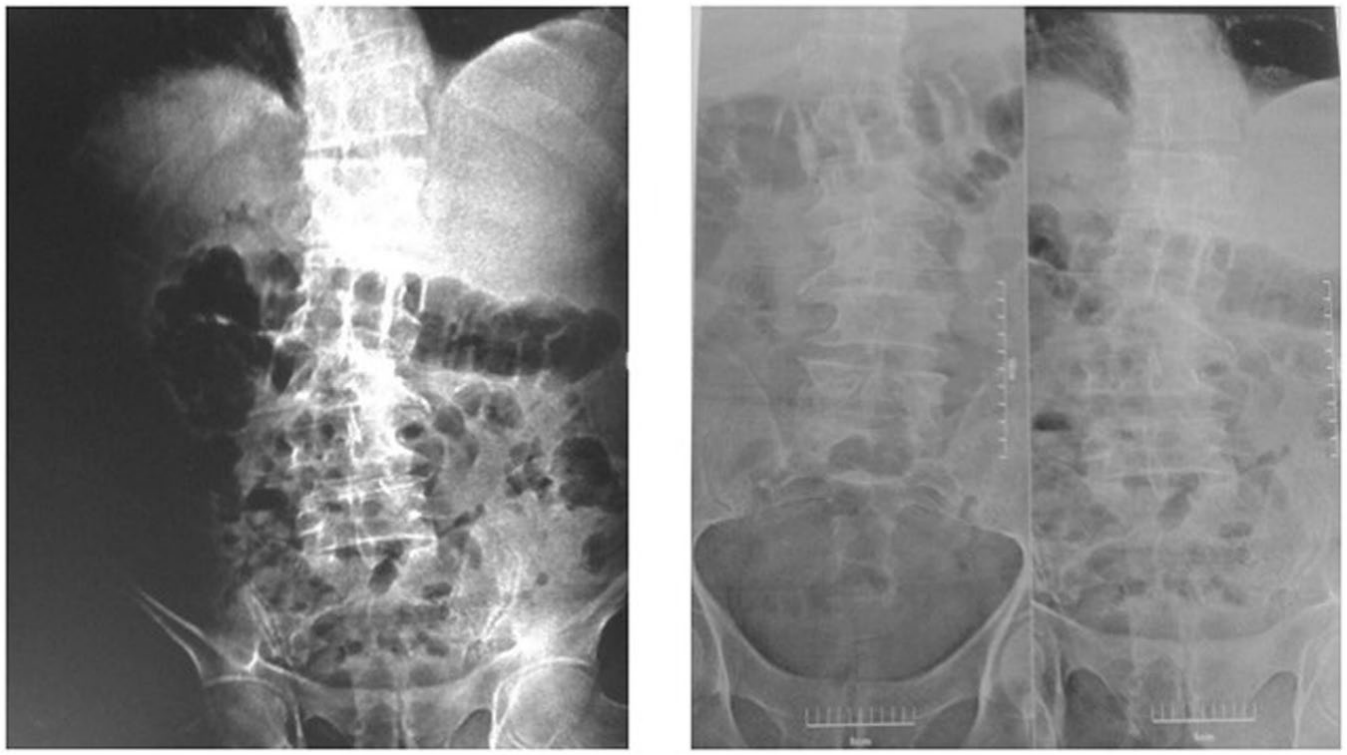
X-ray were referred for bone pain and abnormal findings on radiography.

21.1% of people who live in areas with high fluoride concentrations have bone diseases, which means among the 445 people who live in this region, 94 have skeletal fluorosis (94/445), and 3% of those who live in areas with low fluoride concentration, have skeletal fluorosis were apparent (Fig. 2), which means among the470 people who live in this area, 14 have skeletal fluorosis (14/470). Therefore, it can be said that skeletal fluorosis problem of people who live in areas with high fluoride concentration is 18.1% higher than that of individuals who live in areas with low fluoride concentration. Additionally, the age groups are presented in Table 3. In the case of consumption of milk and dairy, 11.2% of the people who live in these areas (both high and low fluoride) and consume milk and dairy products less than 3 units per week, have skeletal fluorosis and 12.6% of those who consume 3 units and more have skeletal fluorosis (Table 4).

**Table 4:** Distribution of skeletal fluorosis according to sociodemographic characteristics in people.

		Skeletal fluorosis		P
		No	Yes	
Level	High	351 (78.9%)	94 (21.1%)	<0/001*
	Low	456 (97.0%)	14 (3.0%)	
AgeC	<=40	312 (92.0%)	27 (8.0%)	0/027‡
	41–50	262 (87.3%)	38 (12.7%)	
	51–60	158 (89.8%)	18 (10.2%)	
	61–70	65 (83.3%)	13 (16.7%)	
	71+	10 (45.5%)	12 (54.5%)	
Gender	F	391 (85.0%)	69 (15.0%)	0/003*
	M	416 (91.4%)	39 (8.6%)	
Fast food	Never	10 (100.0%)	0 (0.0%)	<0/001‡
	One time in month	71 (74.7%)	24 (25.3%)	
	Two time in month	312 (89.7%)	36 (10.3%)	
	two times in week	414 (89.6%)	48 (10.4%)	
Diary 2	<3	475 (88.8%)	60 (11.2%)	0/513*
	>=3	332 (87.4%)	48 (12.6%)	

^*^Based on Chi-Square test. ^‡^Based on Mann-Whitney test.

According to the 2 Unadjusted* table, people who live in area with low concentration of fluoride have skeletal fluorosis which is 0.12 times higher than people who live in area with high concentration of fluoride. According to the Adjusted **, people who live in area with low concentrations of fluoride have skeletal fluorosis which is 0.11 times higher than that of individuals who live in area with low concentrations of fluoride. The age group of 41–50 years has skeletal fluorosis 2.49 times higher than that of 40 years range, while people who are in the age range of 51–60 have skeletal fluorosis 1.54 times more than people in 41–50 age range. In the case of milk and dairy products, according to Unadjusted*, people who consume ≤3 units milk and dairy products per week have almost the same level of skeletal fluorosis as compared to those who consume 3 units and more. Table 3 also shows that men (Unadjusted*) who live in the high fluoride concentrations area have skeletal fluorosis 11.59 times higher than those who live in low fluoride area, and based on the Adjusted**, men who live in area with high concentrations of fluoride have skeletal fluorosis 6.049 times higher than men who live in low fluoride concentration area, while for women, according to Unadjusted*, those who live in area with high concentrations of fluoride have skeletal fluorosis 8.47 times higher than women who live in area with low concentrations of fluoride, and based on Adjusted**, they have skeletal fluorosis which is 8.727 times higher than that of women who live in area with low concentrations of fluoride (Tables 5, 6).

**Table 5:** The univariate and simultaneous effect of different risk factor on skeletal fluorosis.

		OR	Unadjusted*		P	OR	Adjusted**		
			95% CI				95% CI		P
			Lower	Upper			Lower	Upper	
Level	High	Ref				Ref			
	Low	0.12	0.08	0.18	<0.001	0.11	0.06	0.19	<0.001
AgeC	<=40	Ref				Ref			
	41–50	2.49	1.32	4.68	0.005	1.90	1.36	2.67	<0.001
	51–60	1.54	1.15	2.05	0.004	1.60	1.03	2.49	0.037
	61–70	1.83	1.53	2.18	<0.001	3.17	1.33	7.54	0.009
	71+	12.90	6.25	26.64	<0.001	21.97	11.73	41.16	<0.001
Gender	F	Ref				Ref			
	M	0.56	0.29	1.10	0.094	0.48	0.20	1.14	0.097
Fast food	Never or One time in month	Ref				Ref			
	Two time in month	0.47	0.33	0.69	<0.001	0.61	0.29	1.29	0.195
	two times in week	0.56	0.50	0.62	<0.001	0.79	0.56	1.11	0.176
Diary 2	<3	Ref				Ref			
	>=3	0.98	0.74	1.30	0.898	1.02	0.68	1.53	0.927

^*^Based on simple (univariate) multilevel logistic regression. **Based on Multiple multilevel logistic regression.

Level Vs bone disease:

The rural area had low level of fluoride with odds of 0.12 (95% CI: 0.08 to 0.18, P < 0.001) times as compared to high fluoride region.

Adjusted**

Adjusted for other variables in the model, the rural area with low level of fluoride has odds of 0.11 (95% CI: 0.06 to 0.19, P < 0.001) times as compared to high fluoride region.

**Table 6:** The odds ratio of bone disease in high fluoride versus low fluoride area by gender.

Gender		OR	95% CI		P
			Lower	Upper	
Male	Unadjusted*	11.59	3.54	37.95	<0.001
	Adjusted**	6.049	3.593	10.186	<0.001
Female	Unadjusted*	8.47	4.42	16.24	<0.001
	Adjusted**	8.727	4.311	17.665	<0.001

^*^Based on simple (univariate) multilevel logistic regression. **Based on multiple multilevel logistic regression.

## Discussion and Conclusion

This study revealed that F concentration in drinking water is in the range of 0.22 to 10.33 ppm, minimum and maximum, respectively. Fluoride concentration values of water sources in Poldasht County are higher than the permissible limit (0.5 to 1.5 mg/L) according to WHO guideline^25^ (Table 1). In a series of studies on the various effects of fluoride concentration in drinking water on human tissue, it has been well observed and documented that the toxic effects of fluoride on skeletal system are chronic and occurs over time. Skeletal fluorosis may be displayed by increase in the bone density in adults using X-ray^26,27^ (Fig. 2). The results of this study showed that 21.1% of people who live in areas with high fluoride concentrations have skeletal fluorosis, which means, among the 445 people who live in this region, 94 have skeletal fluorosis (94/445) and 3% of those who live in areas with low fluoride concentration have skeletal fluorosis, meaning that among the 470 people who live in this area, 14 have skeletal fluorosis (14/470) (Table 1). Choubisa showed at 3.2 ppm, 3.7 ppm, and 4.0 ppm water F concentration in villages in India, the highest prevalence of skeletal fluorosis was 39.2, 32.8, and 36.6%, respectively. In the present study, the prevalence of skeletal fluorosis is lower than that reported by this reaserch^28^. Regarding the prevalence of skeletal fluorosis in the two municipalities (3 and 21.4%), these values are within the variation of 31to 80% and 35 to 60% reported for fluoridated communities in the State of São Paulo and United State, respectively^29,30^. Skeletal fluorosis (54.5%) in the age group of 71 years & above was more commonly found in females than males, but the results of a study conducted by Asawa *et al*. on Association of Temporomandibular Joint Signs & Symptoms with Dental Fluorosis & Skeletal Manifestations in Endemic Fluoride Areas of Dungarpur District, Rajasthan, India, are not compatible with those of the present study because Skeletal Fluorosis (61.1%) in the age group of 55–64 years was more commonly found in males than females^31^. Sushella and colleagues in their research found that development of skeletal fluorosis can be due to the consumption of fluoride together with other factors such as low calcium, alkalinity of water, and also, lack of calcium and vitamin C^32^. The present study demonstrates there was significant difference between females and males in the prevalence of skeletal fluorosis (15 and 8.6%) respectively. Watanabe *et al*. showed that the prevalence of skeletal fluorosis was significantly higher for males in a moderately polluted area, but not in a severely polluted area. These results are not consistent with our findings. This matter clearly requires further study^33^. A survey by ChubiSa *et al*. showed that the prevalence of skeletal fluorosis in people who had inappropriate regime increased by 6.61%^34^. Furthermore, study of Karthikeyan *et al*. on Contribution of Fluoride in Water and Food to the Prevalence of Fluorosis in Areas of Tamil Nadu in South India showed a significant correlation between high fluoride concentration in drinking water and enhanced incidence of skeletal fluorosis in 5 fluorine-rich areas, native to Tamilnad^35^.

## Conclusion and Recommendation

Our research is important in that it reports, for the first time in Iran, evidence of fluoride toxicities in people living in the Poldasht County in west Azerbaijan, Iran. The present study demonstrates a significant relationship between the fluoride concentrations in the water and the prevalence of skeletal fluorosis in an endemic fluorosis area. The provision of defluoridated drinking water, and health education aimed at abating fluorosis in people is highly desirable in this village area of Poldasht County in west Azerbaijan, Iran. Government and relevant organizations such as the Department of Energy in the preparation of suitable drinking water in the home and community levels by providing internal filters with affordable costs and the Ministry of Health by raising awareness and appropriate interventions in nutrition, will play important roles in dealing with the problem of fluorosis.
